# Patients’ lived experiences regarding maintaining dignity

**Published:** 2015-04-20

**Authors:** Mohammad Ali Cheraghi, Arpi Manookian, Alireza Nikbakht Nasrabadi

**Affiliations:** 1 *Associate Professor, Faculty of Nursing and Midwifery, Tehran University of Medical Sciences, Tehran, Iran;*; 2 *Assistant Professor, Faculty of Nursing and Midwifery, Tehran University of Medical Sciences, Tehran, Iran;*; 3 *Professor, Faculty of Nursing and Midwifery, Tehran University of Medical Sciences, Tehran, Iran.*

**Keywords:** human dignity, patient rights, qualitative research, hermeneutics, nursing ethics

## Abstract

Preservation of dignity is frequently emphasized as a basic patient’s right in national and international nursing codes of ethics and is indeed the essence and core of nursing care. It is therefore essential to explore the concept based on patients’ lived experiences in order to maintain and respect their dignity and consequently improve the quality of health services and patient satisfaction. The present study aimed to discover the lived experiences of Iranian patients regarding maintaining their dignity at the bedside.

This qualitative study was conducted using an interpretive phenomenological approach. A total of 14 participants (9 women and 5 men) were purposefully selected, and data were collected through individual, semi-structured and deep interviews. The recorded interviews were transcribed and analyzed by the Diekelman, Allen and Tanner approach.

The findings of this study revealed three main themes and related subthemes regarding the meaning of preserving patients’ dignity. The first main theme was “exigency of preserving the innate human dignity” and comprised two subthemes: “respect for the intrinsic equality of all humans” and “treating the patient as a valued person, not an object”. The second theme was “service based on love and kindness” and included two subthemes: ‘being with the patient” and “inspiring the sense of being accepted and loved”. The third main theme emerged as “dignifying and transcendental professional service” and consisted of two subthemes: “professional commitment to uphold patients’ rights” and “enlightened practice”.

This study revealed that the concept of maintaining patients’ dignity is related to health providers’ duty to preserve patients’ dignity and also their moral obligation to manifest the human love that is in their own as well as their patients’ nature. In conclusion, if nurses reflect on the transcendental nature of nursing care, they will value and prize their everyday bedside nursing practice and will utilize their capacities to be more human as well.

## Introduction

Questions regarding the nature of nursing or care are closely linked to ontological questions such as ‘what does it mean to be a person?’ This is why humanistic nursing theories, which have their roots in humanism, existentialism and phenomenological philosophy (1), claim to offer new insights to both patients and healthcare staff and explain the true meaning of humanity, health and nursing (2). From the humanistic point of view, dignity is an intrinsic and objective value that is inherent in every human being (3, 4), and proper nursing care has the potential to bring about further expression of human dignity (4, 5). In this respect, Miller pointed out that care refers to responding to others’ needs by understanding and adopting their ends as one’s own and making an effort to cultivate and restore their agency (5-7). Indeed, providing dignifying nursing care requires fulfilling all dimensions of patients’ physical, emotional, social, cultural and spiritual needs (8). 

In recent years, several quantitative and qualitative studies have been conducted on Iranian patients’ dignity (9,10), but none has adopted a hermeneutic approach to address this phenomenon. The findings of these studies were similar to those of international research in terms of the factors contributing to preservation of patients’ dignity. These factors included: ensuring patient privacy, autonomy and involvement in care, considering patients’ intrapersonal features and beliefs, offering equal and fair treatment, establishing effective communication, providing adequate information and emotional support, and employing proper forms of address (7-11). Many international and national nursing organizations consider the preservation of patients’ dignity to be the essence of nursing care (12-14), and “risk of compromising human dignity” has been listed as an important nursing diagnosis by The North American Nursing Diagnosis Association (15). Nevertheless, preservation of patients’ dignity at the bedside is still a concept not entirely understood. There is a shortage of hermeneutic research on Iranian patients’ experiences in this respect, and the present study was therefore conducted to explore this phenomenon. The hermeneutic approach provides an opportunity to go beyond the popular pre-conceptions through interpretations and an in-depth understanding of the researched phenomenon (16, 17). In this regard, Eriksson noted that regaining the hermeneutic aspect and acquiring a humanistic approach involves surpassing the immediate experiences and getting in touch with the essence of care (3).

## Method

This qualitative study was conducted using Heideggerian hermeneutic phenomenology in order to interpret the lived experiences of patients regarding maintaining their dignity. 

The participants (9 women and 5 men) were purposefully selected from various governmental and non-governmental settings, primarily based on their abilities, their willingness to participate and their experiences regarding the topic of the research. Inclusion criteria for study entry were being over 18 years of age, having the ability to communicate and speak in Persian, willingness to share personal experiences, being oriented in time and place, having at least 48 hours of hospitalization experience. If the patients were ill weak or confused to the extent that would interfere with informed consent or participation in interviews, they were excluded.

They were aged between 30 and 64, and had been hospitalized for a minimum of 2 and a maximum of 30 days. At the beginning of their selection process, they were provided with information about the research objective and ethical considerations. 

The data collection process, which took place between May and November 2012, was completed by the Manookian, who was well acquainted with qualitative interviews. After making arrangements for the appointments, the interviewer visited each patient and created trustful conditions through effective communication, and informed them about the aim of the study and the interview process. 

The participants’ experiences regarding preservation of dignity were elicited using individual in-depth semi-structured interviews structured around the probing questions. They were asked to respond to questions such as: “What is your personal experience of dignified care?” “What does dignified care mean to you?” “What do you understand from the term ‘preserving patients’ dignity’?” and “Can you describe situations when you came across a violation of patients’ dignity?” Moreover, the participants received feedback, and thus verifying and additional data could be obtained during interviews. Each interview took 20 to 60 minutes and was conducted at the participants’ preferred location. In 4 of the cases, more than one interview session was required to reach a comprehensive and deep understanding of the patients’ lived experiences. After each interview, the recordings were carefully listened to several times, immediately transcribed verbatim and analyzed in preparation for the next interview.

***Data Analysis***

The data were analyzed using the Diekelmann, Allen, and Tanner method, which is a seven-step process based on Heidggerian hermeneutic phenomenology. The analysis is typically conducted by an interpretive team and involves seven steps: (i) reading the interviews to obtain an overall understanding; (ii) writing interpretive summaries and coding for emerging themes; (iii) analyzing selected transcripts in groups to identify themes; (iv) returning to the transcribed text or the participants to clarify disagreements in interpretation, and writing a composite analysis for each text; (v) comparing and contrasting the texts to identify and describe shared practices and common meanings; (vi) identifying patterns that link the themes; and (vii) eliciting responses and suggestions on a final draft from the interpretive team and from others who are familiar with the content and methods of the study (18). 

In order to fulfill the above process, the transcribed texts were reviewed several times to gain a general understanding. Then, an interpretive summary was written by all research team members for each interview, and attempts were made to extract hidden meanings. The extracted meaning units from each interview were compared with those from other interviews and were then categorized into subthemes. The process of interpretation was expanded repeatedly by writing and rewriting the transcripts. The research team members read their interpretations and related emerged themes aloud, and the similarities and differences of the interpretations were discussed until a consensus was reached. Subsequently, the main themes and common features of the participants’ experiences were determined. In this way, the continuity of the movement from parts to whole and from whole to parts was observed (16). This interpretive cyclic process continued until the 14th interview where no new meanings emerged and the researchers were satisfied with the depth of their understanding regarding preservation of patients’ dignity at the bedside. 

Several strategies as proposed by Lincoln and Guba (16, 19) were used to enhance the rigor of the findings. Credibility of the findings was established through prolonged contact with the participants and continued engagement with the data during all phases of the research (9 months). Credibility was also ensured through independent analyses conducted by each research team member, and by holding team sessions to reach comprehensive and deep interpretations. It should be noted that the credibility of the researchers was assured by choosing research team members who had sufficient qualifications and were experienced in phenomenological studies. To verify dependability and confirmability, detailed descriptions of the study including evidence and examples were used, so the readers would be able to confirm the findings. The transferability of the findings was assured by (a) offering exact instances and quotes of the participants to enable the readers to compare them with those in similar situations; and (b) providing sufficient contextual information, for instance by choosing participants from both sexes and various age ranges, as well as patients suffering from different medical problems, and from both governmental and non-governmental hospitals. 

***Ethical Considerations***

This research was approved by the ethics review committee of Tehran University of Medical Sciences (approval number 90/130/2691). Participants were given written and verbal information about the purpose of the study and were asked to sign an informed consent. By clarifying the purpose of the study and providing full information on how the data would be used, the research team assured the study subjects that their participation in the study would not impact the quality of the care they received. Moreover, all participants were provided with information about voluntariness of their participation and were assured that their identities would remain confidential during the analysis and reporting of the data to protect their privacy. Ethical considerations were explained to the participants and verbal permission to record the interviews was taken before each interview.

## Results

The findings of the present study revealed that preservation of patients’ dignity involved the following themes: “exigency of preserving the innate human dignity”, “service based on love and kindness” and “dignifying and transcendental professional service”.

*Exigency of preserving the innate human dignity*

The divine origin of the innate human dignity necessitates that all human beings respect each other. Most of the participants placed great importance on “exigency of preserving the innate human dignity”, which consisted of two subthemes: “respect for the intrinsic equality of all humans” and “treating the patient as a valued person, not an object”. 

A 38-year-old man stated: [*I asked them for a new and clean gown, but they ignored my request. I felt that their insensitive treatment of me was due to my social status, and that if I was a patient who came from a big city and had higher economic status, they would treat me in a more respectful way*] (patient No. 1).

A Christian woman described her lived experience regarding the importance of equal treatment of patients and said: [*The doctors and nurses didn’t care if I was Christian or Muslim. They were treating all patients equally*] (patient No. 3).

A dentistry student had an unpleasant experience during hospitalization and said: [*The doctor didn’t even make eye contact with me while I was explaining my problem to him…. This made me feel ignored and devalued, as if I were an inanimate object]* (patient No. 8). 

This theme indicates that people share many of the same human qualities, and preservation of patients’ dignity means to treat them equally, regardless of their gender, position, race and religion. The lived experiences of the participants in this study showed that they expected to be treated not as objects but as human beings who have inherent value. 

*Service based on love and kindness*

According to the findings of this study, preservation of patients’ dignity means having an effective relationship with the patient and providing compassionate care based on love and kindness. In the Iranian context, preservation of patients’ dignity emerged as “service based on love and kindness” including two subthemes: “being with the patient” and “inspiring the sense of being accepted and loved”. 

The subtheme of “being with the patient” comprised two sub-subthemes: “compassion” and “altruism”. The participants pointed out that providing dignified care means being more sensitive to the patients’ holistic needs, especially emotional needs, trying to understand the patients’ feelings, making them feel listened to and supported, and meanwhile showing eagerness to help them to overcome the problems they encounter. Some patients described how they expected their family members to be treated while attempting to express their lived experiences regarding preservation of patients’ dignity. 

A 39-year-old woman stated: [*It’s a wonderful feeling when you need help and they are there promptly, and are eager to meet all your needs … you feel that they are like your close friends or sisters]* (patient No. 4). 

The patients said that they felt valued and respected when they had a sense of being accepted and loved by the nurses. One patient stated: [*They were treating me in such a way that I felt at home and felt like they were my children and had accepted my current state and were loving me in spite of the existing limitations…. Their attitude conveys the massage that you are loved and not just someone who’s imposing an additional burden on them]* (patient No. 7).

Generally speaking, providing dignified care involves empathic and compassionate treatment of the patient. In other words, being with the patient and allowing mutual human love to emerge is a basic requirement in the nursing practice.

*Dignifying and transcendental professional service*

Maintaining patients’ dignity is closely linked to nurses’ responsibility and accountability to provide professional care for patients in order to actualize the inherent values of the nursing profession. The third main theme consisted of two subthemes: “professional commitment to uphold patients’ rights” and “enlightened practice”. The participants pointed out that the health care staff has the responsibility and accountability to observe and advocate patients’ rights. They believed that the staff’s commitment to their professional values helps secure the patients’ rights and preserve their dignity. 

A 35-year-old woman hospitalized for ovarian cyst surgery noted the destructive impact of paternalistic behavior on patients’ dignity and stated: [*When you’re hospitalized, some of the staff (not all of them) keep telling you that you are a patient and you should just accept what is going on. They don’t give you detailed information and don’t respond to your questions and complaints… you just have to keep quiet.… I believe that this is not in line with nursing values…. Being informed of one’s own health status is a basic right of each patient]* (patient No. 5). 

One of the hidden meanings of preserving patients’ dignity was extracted as “enlightened practice”. Indeed, providing dignified care helps nurses to acquire a deeper insight into the value of their practice and highlights the significance of their professional values. Such enlightenment, which is manifested through dignified care, will subsequently lead to a deeper understanding of the value of humanity and promote personal growth in this regard. 

A 43-year-old woman who was also a nurse hospitalized in a medical-surgical ward said: [*To me,* *preserving patients’ dignity is possible when nurses’ self-awareness is raised through responding to patients' needs while providing care. If we reflect on the value of our everyday nursing practice, we will discover the core value of our profession, which is nothing but preserving patients’ dignity…. During my hospitalization one of my colleagues pulled the bed sheet I was lying on so carelessly that my urinary catheter was pulled out, and that caused terrible damage to my urinary tract... if she had thought about the value of care, she never would have acted in such a carless way]* (patient No. 6). 

Generally our findings emphasized the importance of recognizing and preserving patients’ dignity through providing professional nursing care and preparing the ground to actualize the core values of the nursing profession.

## Discussion

This study revealed that Iranians have different notions of preserving patients’ dignity at the bedside. There have been numerous attempts in health and social care literature to define patients’ dignity preservation, but no explicit definition has been offered for this phenomenon so far. The reason might well be the subjectivity and complexity of this concept that leads to various perceptions of dignity preservation by different people (20-23). Manookian et al. pointed out that human dignity is influenced by personal, cultural, social, religious, and spiritual constructs and contexts, and must therefore be interpreted based on people’s perspectives and lived experiences under specific circumstances (24). It is extremely important for healthcare professionals to comprehend the true meaning of maintaining patients’ dignity in order to provide effective and dignified care for patients, who have inherent worth endowed with divine blessing (20, 23, 25). The inherent value of human beings has been emphasized in a verse in the Quran “and we have certainly honored the children of Adam…” (26). 

In the present study, the first main theme emerged as “exigency of preserving the innate human dignity”. This is in line with numerous studies conducted in different contexts, which have highlighted the necessity of respecting each patient’s intrinsic worth as a core value of the nursing profession (22-29). It seems that the above-mentioned theme encompasses the concept of obligation as well. In this respect, Nightingale’s valuable point of view regarding the nature of the nursing profession should be noted. She reminds us that we are accountable to God for respecting the dignity of each human being in this world (30, 31). In other words, the exigency of preserving patients’ dignity could be discussed from the ontological perspective. Accordingly, it is absolutely essential to establish an appropriate relationship with patients and respond to their demands through caring for them and by attending to their individual needs. This will prepare the ground for recognizing and acknowledging the human worth of both patients and nurses, as well as their uniqueness and individuality. Affirmation of this equal inner worth reminds us of our obligation to respond to each other’s needs through caring, which can lead to enhanced well-being and consequently promote dignity (5, 32). This ontological perspective can be extended to the second main theme of the present study, that is, “service based on love and kindness”. According to Paterson and Zderad’s humanistic nursing theory, nursing care, whose ultimate goal is to enhance people’s well-being, is a special kind of nurse-patient intersubjective transaction that offers the ground for personal development through the nurturing response of one person to another in need. This humanistic response occurs through the active presence of the nurse in time and space that is known as “being with the patient” (33, 34). Through this authentic presence, mutual human love flows between the spirits of two human beings and actualizes the true meaning of nursing care. 

Findings of the present study were in keeping with various research (8, 20-29, 35, 36) pointing to the significance of compassion and emotional support in maintaining and promoting patients’ dignity. Indeed, caring is a human-to-human relationship and is therefore contingent on compassionate behavior (2) and emotional involvement as well as taking note of one’s own feelings, experiences, and reactions (37). Olshansky pointed out that in nursing practice, it is essential to remember the fundamentals of service based on love and compassion and above all return to the art of caring, which tends to be marginalized nowadays (38). 

According to the participant’s lived experiences, the third theme was extracted as “dignifying and transcendental professional service”, which included two subthemes: “professional commitment to uphold patients’ rights” and “enlightened practice”. In this regard, Heijkenskjöld et al. found that failure to dedicate personal time to patients, acting indifferently towards them and regarding them as objects who are not allowed to participate in their own care are instances of non-professional behavior that could compromise patients’ dignity (39). Other studies have also shown that patients’ dignity will be compromised if nurses do not take professional responsibility to advocate patients’ rights. Cases in point include paternalistic interactions with patients (11, 37), offering treatment in a matter-of-fact way (36), disregard for patients’ privacy (11, 21, 22, 27, 28) and their personal, religious and cultural values (25).

“Enlightened practice” was the second subtheme that emerged as a hidden meaning of preserving patients’ dignity based on the participants’ lived experiences. In this regard, providing dignified care offers an opportunity of self-reflection for the nurses to think about the values underlying their practice. Buckley also found that treating patients with dignity could have a positive effect on caregivers since they affirm their own dignity through establishing the value of the care they provide (32). However, it seems that authentic care implies unconditional respect for others and fulfillment of their needs, which is nothing but the definition of humanity. Whenever a nurse and a patient come together in a human-to-human interaction, they both have the possibility to affect and be affected, that is, to become more human. Indeed, acknowledgment of patients’ dignity provides the nurses with the opportunity to promote their self-awareness, personal growth and wisdom, which can in turn help them acquire further insight into their professional identities and healing capacities (31-34). Watson called this a “caring moment”, whereby both the nurse and the patient feel a deep spiritual connection by transcending time and space, and each recognize their human dignity in the other and attempt to proceed on the caring-healing-loving journey (40).

The present study was conducted to provide a deeper understanding of Iranian patients’ experiences regarding maintaining their dignity and consequently their feelings and expectations. The findings may be used to develop practical guidelines and formulate more specific strategies in this regard. Moreover, this study addressed a shortage of knowledge at the international level and could be applied worldwide and in diverse clinical settings, especially in contexts with a strong religious belief.

## Conclusion

Preservation of patients’ dignity is a central phenomenon in nursing practice and nurses have a professional obligation to maintain and promote patients’ dignity. The present study explored the meaning of dignity preservation based on Iranian patients’ lived experiences. The nurses’ obligation to preserve their patients’ dignity could be discussed on the grounds of the divine origin of the innate human dignity and the equality of the patient and the nurse. Accordingly, acknowledging the equal inner worth of patients forms the basis for providing care based on love and kindness. Indeed, through this type of care, mutual human love flows between the spirits of two human beings and actualizes the true meaning of nursing care. It seems that the current crisis in the nursing profession is due to a lack of centralization and reflection on some ontological concepts such as transcendental nursing care, which can promote dignifying care and at the same time prepare the ground for personal and professional growth. In other words, if nurses reflect on the transcendental nature of providing professional dignifying care for the patients, they will value and prize their everyday bedside nursing practice more and will better utilize their capacities to be more human as well.

Although the Ministry of Health and Medical Education of Iran has emphasized the importance of implementing a professional code of ethics and respect for patients’ rights, there are still issues that need to be addressed in this regard. In addition to managerial and practical areas, the findings of this study may benefit nursing ethics education in undergraduate, postgraduate and continuing education programs.

## References

[1] Paterson JG, Zderad LT (1976). Humanistic Nursing.

[2] Watson J (2011). Human Caring Science: A Theory of Nursing.

[3] Eriksson K (2002). Caring science in a new key. Nurs Sci Q.

[4] Sensen O (2011). Kant on Human Dignity.

[5] Miller SC (2012). The Ethics of Need: Agency, Dignity and Obligation.

[6] Gallagher A (2004). Dignity and respect for dignity-two key health professional values: implications for nursing practice. Nurs Ethics.

[7] Burns L ( 2008). What is the scope for the interpretation of dignity in research involving human subjects?. Med Health Care Philos.

[8] Gastmans Ch (2013). Dignity-enhancing nursing care: a foundational ethical framework. Nurs Ethics.

[9] Nayeri ND, Karimi R, Sadeghee T (2011). Iranian nurses and hospitalized teenagers’ views of dignity. Nurs Ethics.

[10] Ebrahimi H, Torabizadeh C, Mohammadi E, Valizadeh S (2012). Patients’ perception of dignity in Iranian healthcare settings: a qualitative content analysis. J Med Ethics.

[11] Baillie L (2009). Patient dignity in an acute hospital setting: a case study. Int J Nurs Stud.

[12] Zahedi F, Sanjari M, Aala M (2013). The code of ethics for nurses. Iran J Public Health.

[13] Anonymous The code: professional standards of practice and behavior for nurses and midwives. https://www.nmc.org.uk/globalassets/sitedocuments/nmc-publications/nmc-code.pdf.

[14] Anonymous Code of ethics for nurses with interpretive statements. http://www.nursingworld.org/MainMenuCategories/EthicsStandards/CodeofEthicsforNurses/Code-of-Ethics-For-Nurses.html.

[15] Anonymous Nursing diagnosis—risk for compromised human dignity. http://nandanursingdiagnosis.org/nursing-diagnosis-risk-compromised-human-dignity/%20.

[16] Holloway I, Wheeler S (2010). Qualitative Research in Nursing and Healthcare.

[17] Benner PE (1994). Interpretive Phenomenology: Embodiment, Caring, and Ethics in Health and Illness.

[18] Dikelman NL (2003). Teaching the Practitioners of Care. New Pedagogies for the Health Professions.

[19] Streubert Speziale H, Streubert HJ, Carpenter DR (2011). Qualitative Research in Nursing: Advancing the Humanistic Imperative.

[20] Griffin-Heslin VL (2005). An analysis of the concept dignity. Accid Emerg Nurs.

[21] Haddock J (1996). Towards further clarification of the concept dignity. J Adv Nurs.

[22] Baillie L, Gallagher A (2011). Respecting dignity in care in diverse care settings: strategies of UK nurses. Int J Nurs Pract.

[23] Rogers-Clark C, McCarthy A, Martin-McDonald K (2005). Living with Illness, Psychosocial Challenges for Nursing.

[24] Manookian A, Cheraghi MA, Nasrabadi AN (2014). Factors influencing patients' dignity: a qualitative study. Nurs Ethics.

[25] Cheraghi MA, Manookian A, Nasrabadi AN (2014). Human dignity in religion-embedded crosscultural nursing. Nurs Ethics.

[26] The Noble Quran.

[27] Matiti MR, Trorey GM (2008). Patients' expectations of the maintenance of their dignity. J Clin Nurs.

[28] Lin YP, Tsai YF, Chen HF (2011). Dignity in care in the hospital setting from patients' perspectives in Taiwan: a descriptive qualitative study. J Clin Nurs.

[29] Shahriari M, Mohammadi E, Abbaszadeh A, Bahrami M, Fooladi M (2012). Perceived ethical values by Iranian nurses. Nurs Ethics.

[30] Williams R Nightingale not an angel: archbishop of canterbury.

[31] Watson J (2009). Caring science and human caring theory: transforming personal and professional practices of nursing and health care. J Health Hum Serv Adm.

[32] Buckley Sh Patient Dignity: the Significance of Relationship [dissertation].

[33] Meleis AI (2005). Theoretical Nursing: Development and Progress.

[34] O’Connor N (1999). Paterson and Zedrad: Humanistic Nursing Theory.

[35] Whitehead J, Wheeler, H (2008). Patients’ experiences of privacy and dignity. Part 1: a literature review. Br J Nurs.

[36] Henderson A, Van Eps MA, Pearson K, James C, Henderson P, Osborne Y (2009). Maintenance of patients’ dignity during hospitalization: comparison of staff-patient observations and patient feedback through interviews. Int J Nurs Pract.

[37] Hanssen I, Alpers LM (2010). Utilitarian and common-sense morality discussions in intercultural nursing practice. Nurs Ethics.

[38] Olshansky E (2007). What do we mean by compassion and caring in nursing and why does it matter anyway. J Prof Nurs.

[39] Heijkenskjöld KB, Ekstedt M, Lindwall L (2010). The patient's dignity from the nurse's perspective. Nurs Ethics.

[40] Watson J (2003). Love and caring. Ethics of face and hand—an invitation to return to the heart and soul of nursing and our deep humanity. Nurs Adm Q.

